# Supplementary material for: MetaQTL: a package of new computational methods for the meta-analysis of QTL mapping experiments
Source: BMC Bioinformatics. 2007 Feb 8;8:49. doi: 10.1186/1471-2105-8-49 (PMC1808479; doi:10.1186/1471-2105-8-49)
Supplement: Additional File 7 — MetaQTL Package : jar file and tutorial. This Zip archive contains both the MetaQTL JAR file and the files of the tutorial. [file 1471-2105-8-49-S7.zip › org.inra.metaqtl/doc/org/metaqtl/class-use/MetaQtlResult.html]

Uses of Class org.metaqtl.MetaQtlResult


|  |  |  |
| --- | --- | --- |
| **Overview** \| **Package** \| **Class** \| **Use** \| **Tree** \| **Deprecated** \| **Index** \| **Help** | |  |
| PREV   NEXT | **FRAMES**    **NO FRAMES**     **All Classes** |  |


---


## **Uses of Class org.metaqtl.MetaQtlResult**

| Packages that use MetaQtlResult | |
| --- | --- |
| **org.metaqtl** |  |
| **org.metaqtl.adapter** |  |
| **org.metaqtl.algo** |  |

Uses of MetaQtlResult in org.metaqtl

| Fields in org.metaqtl declared as MetaQtlResult | |
| --- | --- |
| `MetaQtlResult[]` | `Chromosome.metaQtlAnalysis`             The results of a meta-analysis of QTL. |
| `MetaQtlResult[][]` | `MetaQtlAnalysis.resultByChrom`             The matrix of results where the rows are the chromosomes. |

| Methods in org.metaqtl that return MetaQtlResult | |
| --- | --- |
| `MetaQtlResult` | `MetaQtlAnalysis.getResult(int chromIdx, java.lang.String trait)`             Returns the result for the given trait name or null if not found. |
| `MetaQtlResult` | `MetaQtlAnalysis.getResult(java.lang.String chrom)`             Returns the first result for the given chromosome. |
| `MetaQtlResult` | `MetaQtlAnalysis.getResult(java.lang.String chrom, java.lang.String trait)`             Returns the result for the given trait name or null if not found. |
| `MetaQtlResult[]` | `MetaQtlAnalysis.getResults(java.lang.String chromName)` |

Uses of MetaQtlResult in org.metaqtl.adapter

| Methods in org.metaqtl.adapter with parameters of type MetaQtlResult | |
| --- | --- |
| `static QtlPartition` | `QtlPartitionAdapter.adapt(MetaQtlResult result, Qtl[] qtls, int k)` |

Uses of MetaQtlResult in org.metaqtl.algo

| Methods in org.metaqtl.algo that return MetaQtlResult | |
| --- | --- |
| `MetaQtlResult[]` | `QtlTreeAlgorithm.buildQtlTree(MetaQtlData data)`             For the given data points this method build the tree which represents the hierarchical clustering. |
| `MetaQtlResult[]` | `QtlClustAlgorithm.doQtlClustering(MetaQtlData data)`             For a given data set `data` performs the clustering on each trait group and returns the results as an array of `MetaQtlResult` objects. |

---


|  |  |  |
| --- | --- | --- |
| **Overview** \| **Package** \| **Class** \| **Use** \| **Tree** \| **Deprecated** \| **Index** \| **Help** | |  |
| PREV   NEXT | **FRAMES**    **NO FRAMES**     **All Classes** |  |


---
